# Investigation of Catalytic Co-Pyrolysis Characteristics and Synergistic Effect of Oily Sludge and Walnut Shell

**DOI:** 10.3390/ijerph20042841

**Published:** 2023-02-06

**Authors:** Qinghong Li, Huan Yang, Ping Chen, Wenxue Jiang, Fei Chen, Xiaorong Yu, Gaoshen Su

**Affiliations:** 1 School of Chemical and Environmental Engineering, Yangtze University, Jingzhou 434023, China; 2 Drilling and Production Engineering Technology Research Institute of CNPC Chuanqing Drilling Engineering Co., Ltd., Chengdu 710018, China; 3 National Engineering Laboratory for Exploration and Development of Low Permeability Oil and Gas Fields, Xi’an 710018, China; 4 CCDC Chuangqing Downhole Technology Company, Xi’an 710018, China

**Keywords:** oily sludge, co-pyrolysis, biomass, ZSM-5

## Abstract

The co-pyrolysis of oily sludge and walnut shell is a reliable method for solid waste treatment and waste recycling. In this paper, a thermogravimetric analysis was used to study the thermodynamics and synergy effect of oily sludge (OS) and walnut shell (WS) at four heating rates (10, 20, 30, and 40 °C/min) in the temperature range from 50–850 °C. Two model-free methods (FWO and KAS) were used to calculate the activation energy. The results showed that the heating rate had no significant effect on the pyrolysis process. The addition of walnut shell improved the pyrolysis process of the samples. Mixture 1OS3WS had a synergy effect, while other blends showed an inhibitory effect. The synergy effect of co-pyrolysis was strongest when the mass ratio of oily sludge was 25%. The activation energy of the Zn-ZSM-5/25 catalyst was the lowest, and the residual substances were the least, indicating that the Zn-ZSM-5/25 was beneficial to the co-pyrolysis of oily sludge and walnut shell. The analysis of catalytic pyrolysis products by Py-GC/MS found that co-pyrolysis was beneficial to the generation of aromatic hydrocarbons. This study provided a method for the resource utilization of hazardous waste and biomass waste, which was conducive to the production of aromatic chemicals with added value while reducing environmental pollution.

## 1. Introduction

Oily sludge (OS) is one of the main pollutants in the petroleum and petrochemical industry [1]. According to recent statistics, the historical inventory of OS in China has reached 143 million tons and is increasing at the rate of 5 million tons per year [2]. The composition of OS is complex, including radioactive substances, pathogenic microorganisms, harmful elements, and organic matters. Total petroleum hydrocarbons (TPH) have a strong adsorption capacity to soil, and the gas released due to the action of microorganisms is extremely harmful to environment and human health [3,4]. Meanwhile, the high oil content and high calorific value of OS indicate that it has a huge energy potential [5,6]. Considering the energy demand and environmental protection, the ultimate goal of OS treatment is to immobilize the hazardous elements in the residue and to improve crude oil utilization. Among the various OS treatment technologies, pyrolysis is the most suitable method for achieving this goal [7]. Although pyrolysis technology has been previously reported to have an excellent ability in the reduction of oil content, it is difficult to ensure that the heavy oil fraction in OS meets the standard just by raising the temperature. Asphaltenes and carboxylic acids in heavy oil fractions can seriously affect the quality of pyrolysis oil. Adding catalysts to co-pyrolysis with other substances is an effective way to achieve these goals [8,9,10].

Due to the characteristics of a large quantity and sufficient source, renewable biomass can be an effective alternative to fossil energy [11]. However, its disadvantages, such as high oxygen content, low calorific value, poor thermal stability, and strong corrosivity, hinder the practical application of bio-oil in industrial production and daily life [12,13]. To solve this problem, co-pyrolysis of biomass with OS is undoubtedly a good method. Co-pyrolysis of biomass with OS not only solves the problem of waste treatment and resource utilization but also is an important technical way to improve the quality of oil products. Oily sludge tends to crack at a high temperature, and its pyrolytic oil has a high hydrogen content and high calorific value. Biomass has a low cracking temperature, and the bio-oil produced by pyrolysis has lower hydrogen content and lower calorific value. Therefore, the co-pyrolysis of biomass and OS is a potential method to produce high-quality oil and gas [14].

In order to further improve the quality of pyrolytic oil, catalysts are usually used [15]. Because of its unique topological structure, a zeolite molecular sieve can be used as an efficient catalyst [16,17]. The Bronsted acid site of zeolite can catalyze cyclization and hydrogen transfer reactions. Due to perfect deoxidation and hydrocarbon selectivity with high value added, HZSM-5 has proven to be the most efficient catalyst for the production of aromatics. HZSM-5 has a large number of acidic sites, which can promote the generation of aromatic hydrocarbons and coke. However, the generated coke will accumulate in the pore, resulting in the reduction of the service life of the catalyst [18,19,20]. When transition metal ions are introduced into zeolite catalysts, aromatization can occur at the center of metal Lewis acid through dehydrogenation rather than hydrogen transfer, thus forming molecular hydrogen as a byproduct and increasing the service life of the catalyst [21,22]. Previous studies have shown that metallic Zn can form Zn sites in zeolite and promote aromatization by dehydrogenation of additional sites, thus promoting pyrolysis of biomass [17].

Therefore, in this study, a Zn-modified HZSM-5 catalyst was co-pyrolyzed with oil sludge and walnut shell (WS). The influence of different proportions and catalysts on the pyrolysis reaction was investigated by a thermogravimetric analyzer (TGA). The activation energy of the sample was studied by two model-free methods (FWO and KAS). The pyrolysis products were analyzed by Py-GC/MS. The results were used to analyze the possible synergistic interaction and catalytic co-pyrolysis mechanism between the two components under different catalyst conditions.

## 2. Experiment Materials and Methods

### 2.1. Materials

The raw material used in the experiment was oily sludge (OS), which was taken from an oilfield in Qianjiang. The biomass was walnut shell (WS) from the Yunnan region. Before the experiment, the oily sludge and walnut shells were dried in an oven at 105 °C for 12 h, followed by crushing and sieving to obtain particles less than 75 μm and stored in sealed bags for later analysis. The physicochemical analysis of oily sludge and walnut shell were analyzed, and the results are listed in Table 1. The zeolite powders HZSM-5/25, HZSM-5/110, HZSM-5/500, and Zn(CH_3_COO)_2_ used in this experiment were purchased from Shanghai Aladdin Technology Co., Ltd. (Shanghai, China).

### 2.2. Preparation of Catalysts

First, a 0.2 mol/L Zn(CH_3_COO)_2_ solution was added to the three molecular sieves, and reflux was conducted at 80 °C for 12 h. The samples obtained after the reaction were centrifuged, washed (pH = 6), and dried in the oven at 110 °C for 12 h. Finally, the dried samples were calcined at 550 °C for 5 h before use. The content of the catalyst elements was obtained by inductive coupled plasma emission spectrometer (Agilent 720ES, Santa Clara, CA, USA). The results are listed in Table 2.

### 2.3. Characterization of Samples

The structure and chemical bonds of the sample were characterized using the Fourier transform infrared spectrometer (Nicolet 6700, Thermo Fisher Scientific, Waltham, MA, USA). The scanning range was 4000–700 cm^−1^, and the scanning times were 32 times

The crystal structure of the catalyst was analyzed by powder X-ray diffractometer (Panaco EMPYREAN, Almelo, The Netherlands). The instrument used a Cu target to emit radiation with incident wavelength of 0.15 nm, tube voltage of 40 kV, and tube current of 40 mA. Continuous scanning was performed in the range 2θ = 5–50° at a rate of 5/min.

### 2.4. Thermogravimetric Experiment

Thermogravimetric experiments were performed using a thermogravimetric analyzer (SETARAM, Lyon, France) to evaluate the pyrolysis characteristics of the sample. According to previous reports, most biomass pyrolysis occurs between 220–520 °C. The volatilization temperature of light and heavy components of OS occurred at 240–550 °C and 700–850 °C, respectively. We chose four different heating rates for the experiments so that the activation energy could be calculated by the equal conversion method [7,16]. Thus, the sample was heated from 50 °C to 850 °C at four heating rates (10, 20, 30, and 40 °C/min). The nitrogen flow rate was 30 mL/min, and the weights of samples were about 8 mg. The mixing ratio of oily sludge and walnut shell were 1:0, 3:1, 1:1, 1:3, and 0:1 (respectively described as OS, 3OS1WS, 1OS1WS, 1OS3WS, and WS). Three Zn-modified catalysts were added to the mixture of sludge and walnut shells, which were described as 1OS3WS + C1, 1OS3WS + C2, and 1OS3WS + C3.

### 2.5. Kinetic Parameter Analysis

In the process of pyrolysis kinetics analysis of solid phase system, the multiple scanning rate method is a relatively common method. Through the data at the same conversion rate on multiple TG curves were measured at different heating rates, a relatively reliable activation energy value can be obtained without involving the kinetic model [23]. In the process of solid pyrolysis analyzed by thermogravimetric method, the kinetic equation under non-isothermal conditions is as follows:

(1) $$\frac{d\alpha }{dt}=\frac{A}{\beta }\cdot e^{\left(\frac{-E}{RT}\right)}\cdot f\left(\alpha \right)$$

where *α*, *β*, *A*, *R*, *E*, and *T* represent the conversion rate (%), the heating rate (K/min), the pre-exponential factor, the gas constant (8.314), the activation energy (kJ/mol), and the reaction temperature (K), respectively. *f*(*α*) is the mechanism function in a differential form.

The conversion rate is calculated using the following expression:

(2) $$\alpha =\frac{m_{0}-m_{t}}{m_{0}-m_{f}}$$

where *m*_0_, *m_t_*, and *m_f_*, are the initial sample mass, the sample mass at time “*t*”, and the residue mass at the end of the reaction, respectively.

The data processing methods of the TG curve can be divided into the integral method and differential method. The integral method is represented by Flynn–Wall–Ozawa (FWO) method, and the differential rules is represented by the Kissinger–Akahira–Sunose (KAS) method [24,25,26]. In order to avoid using the data measured from a single heating rate to evaluate the activation energy and other kinetic parameters, we used the above two methods. However, there are still some limitations in the use of these two methods. Their accuracy is not high, and they are limited to linear heating rate conditions [27,28]. On the premise that no mechanism function is assumed, the integral method and differential method can improve the reliability of analysis results. The equations of the FWO and KAS methods can be expressed as Equations (3) and (4), respectively.

(3) $$\ln\left(\frac{\beta }{T^{2}}\right)=\ln\frac{AR}{EG\left(\partial \right)}-\frac{E}{RT}$$

(4) $$\ln\beta =\ln\frac{AR}{EG\left(\partial \right)}-5.3305-1.052\frac{E}{RT}$$

If the same α is selected for different *β*, the mechanism function *G*(*α*) in the integral form is a constant value, so ln(*β*/*T*^2^) and ln*β* have a linear relationship with 1/*T*, and *E* can be obtained from its slope.

### 2.6. Py-GC/MS Experiment

In this study, the pyrolytic part (Pyroprobe5200, CDS Analytical LLC, Oxford, PA, USA) and GC/MS Online detection section (GC/MS-OP2010, Shimadzu, Kyoto, Japan) were connected to complete this experiment. The test column was methylpolysiloxane (5% phenyl). A Py-GC/MS experiment was used to analyze volatile organic compounds and other components by combining gas chromatograph and mass spectrometer. In each group of experiments, 0.5 mg samples were taken and loaded into a quartz tube. The quartz tube containing the sample was placed into a cracking device for the cracking test and analysis. In this experiment, the oily sludge and walnut shell were co-pyrolyzed with different catalysts to analyze the types and distribution of organic matter produced by the final pyrolysis of the mixture with different catalysts. The composition of pyrolysis products was analyzed according to the NIST spectrum and Wiley spectrum.

## 3. Results and Discussion

### 3.1. Characterization of Samples

#### 3.1.1. FT-IR Analysis

**Figure 1:**
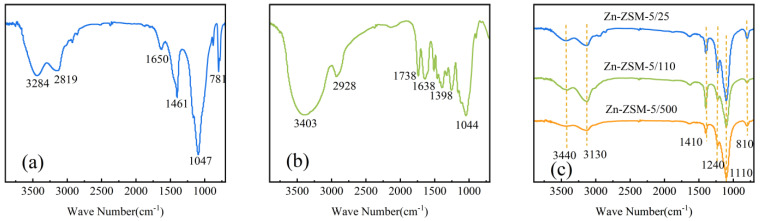
FTIR spectra of (**a**) OS, (**b**) WS, and (**c**) catalysts.

The infrared spectrum of oily sludge is shown in Figure 1a. As can be seen from the figure, the bands at 3284 cm^−1^ and 2819 cm^−1^ correspond to the C-H bond stretching vibration on unsaturated carbon and saturated carbon respectively. The band at 1650 cm^−1^ is due to the stretching vibration of the C=C bond. The peaks at 1461 cm^−1^ and 1047 cm^−1^ are attributed to the stretching vibrations in and out of the C-H plane. The band at 781 cm^−1^ may be due to intermediate substitution of benzene ring [11].

The infrared spectrum of the biomass is shown in Figure 1b. It can be observed that biomass is composed of a variety of oxygen-containing functional groups. The band at 3403 cm^−1^ can be attributed to the stretching vibration of hydroxyl, and that at 2928 cm^−1^ can be attributed to the C-H stretching vibration of methyl and methylene. The peaks at 1738 cm^−1^ and 1638 cm^−1^ correspond to the stretching vibration of unconjugated and conjugated C=O bonds respectively, and the peaks at 1398 cm^−1^ and 1044 cm^−1^ are due to the bending vibration of C-H bonds and the out-of-plane deformation vibration of C-H aromatic ring, respectively.

The infrared spectrum of the catalyst is shown in Figure 1c. It can be seen that the absorption bands at 1110 cm^−1^ and 810 cm^−1^ correspond to the asymmetric and symmetric Si-O-Si stretching vibration peaks of silica [29]. The band at 1410 cm^−1^ is the characteristic absorbing vibration peak of alumina.

#### 3.1.2. XRD Analysis

**Figure 2:**
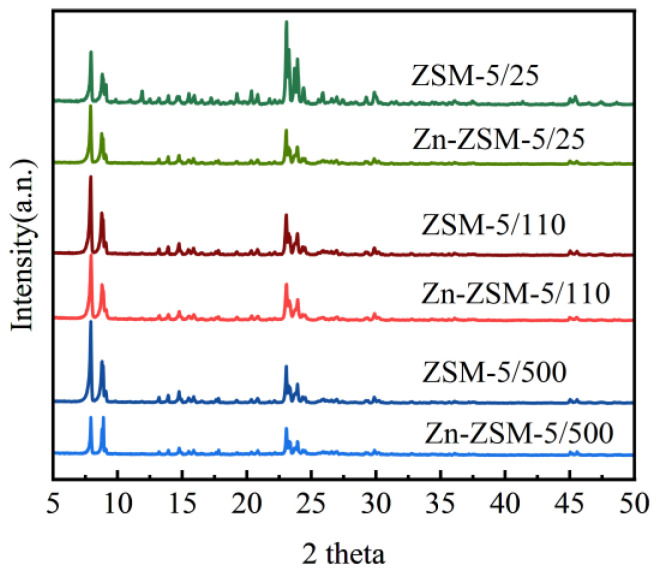
XRD plot of the effect of zinc modified ZSM-5 zeolite.

The XRD pattern of the ZSM-5 molecular sieve before and after modification are shown in Figure 2. As shown in the figure, most of the samples showed typical MFI structural peaks at 2θ values of 7.92°, 8.87°, 23.08°, 23.92°, and 24.34°, indicating that the samples have high crystallinity. There was no obvious characteristic peak of zinc morphology in the figure, indicating that zinc was distributed in the catalyst evenly and no large particles were formed. The zinc modification treatment had no effect on the framework structure of the molecular sieve.

### 3.2. Thermogravimetric Analysis of Samples

#### 3.2.1. Effect of Heating Rate on Pyrolysis Characteristics

**Figure 3:**
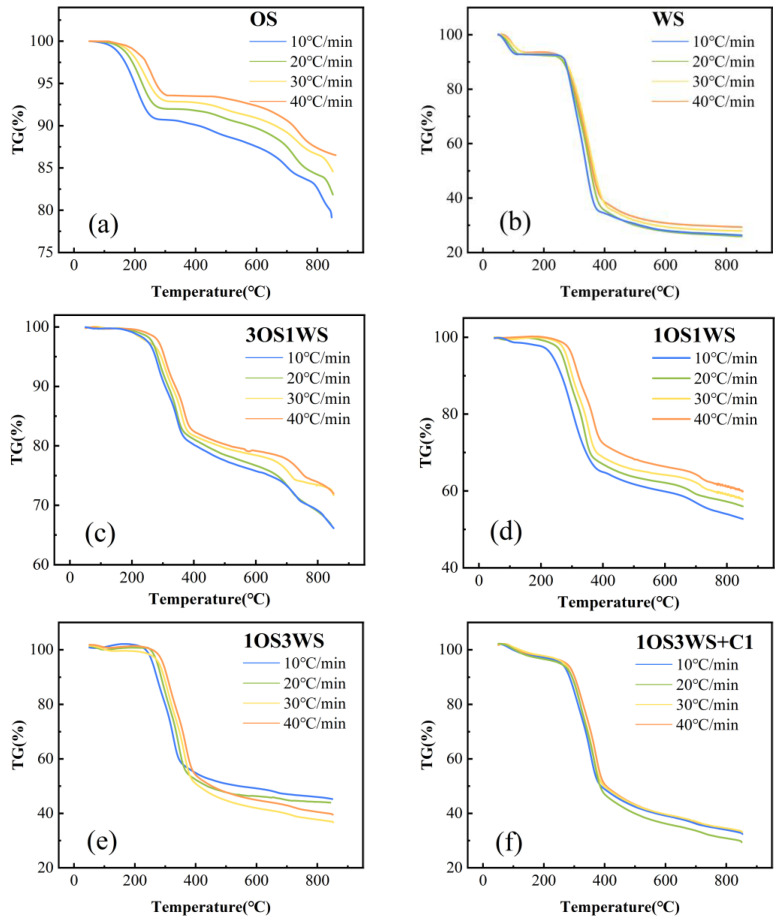

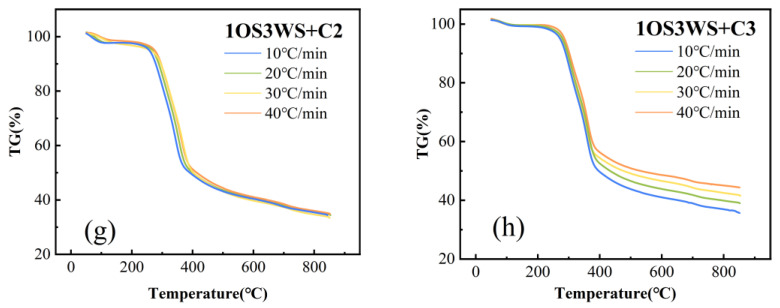
TG curves of (**a**) OS, (**b**) WS, (**c**–**e**) their mixtures, and (**f**–**h**) mixture with catalysts at different heating rates.

The TG and DTG curves of oily sludge (OS), walnut shell (WS), their mixture, and mixture with catalyst at four different heating rates (10, 20, 30, and 40 °C/min) are shown in Figure 3 and Supplementary Figure S1, respectively. As can be seen from the figure, different heating rates had no influence on the whole pyrolysis process. In most cases, the maximum weight loss was achieved at a heating rate of 10 °C/min, which may be due to the fact that the sample is heated uniformly and reacts more completely at a lower heating rate, while thermal hysteresis may occur at a higher heating rate [30]. As can be seen from Supplementary Figure S1, with the increase of heating rate, the initial decomposition temperature, termination temperature, and maximum weight loss rate of the sample all moved towards high temperature, and the maximum mass loss rate gradually increased. Although the heat loss rate is usually the highest at 40 °C/min, the most samples are involved in the reaction. However, at this heating rate, some subtle curve changes are masked, which may be caused by thermal lag. Therefore, considering the thermal loss rate and thermal lag of the sample, the data with a heating rate of 30 °C/min was selected for further discussion [31,32].

#### 3.2.2. Effect of Mixing Ratio on Pyrolysis Characteristics

**Figure 4:**
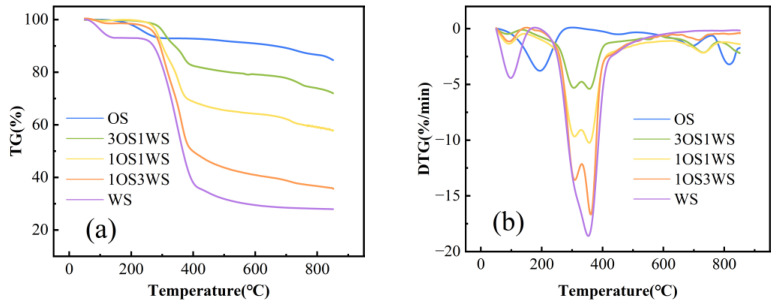
TG curve (**a**) and DTG curve (**b**) of oil sludge and walnut shell at different mixing ratios.

The TG and DTG curves of OS, WS and their mixtures at the heating rate of 30 °C/min are shown in Figure 4, and the main characteristic parameters are shown in Table 3. Peaks at temperatures below 100 °C will not be discussed in the following discussion as they were mainly due to the evaporation of water. As shown in Figure 4, the pyrolysis process of oily sludge can be divided into three stages. The first stage (101–292 °C) was mainly the volatilization of light components in oily sludge. The second stage (316–537 °C) may correspond to the decomposition of heavy components by heat. The third stage (617–775 °C) may be caused by the decomposition of inorganic carbonates [31]. Different from oily sludge, the pyrolysis process of walnut shell showed only one weight loss stage, which was centered at 352 °C. The weight loss at this stage was mainly caused by the decomposition of three components. The thermal properties of hemicellulose were less stable, and decomposition occurred first, followed by cellulose decomposition. There was only one peak in the DTG curve, which was probably due to the low crystallinity of the cellulose in the walnut shell. Due to the stable nature of lignin, the weight of walnut shell continued to decrease after 380 °C, which was caused by the deep pyrolysis and carbonization of lignin [11,33]. Compared with the pyrolysis of raw materials alone, the co-pyrolysis of mixtures showed a more complex pyrolysis process. The peaks of the mixture in the first stage were similar to those of walnut shells, indicating that the weight loss at this stage was mainly caused by walnut shell pyrolysis. It can be clearly seen from the figure that the initial reaction temperature of the mixture moved backward, and the walnut shell softened to different degrees and covered the surface of the oily sludge, which hindered the release of volatiles from the oily sludge.

#### 3.2.3. Effect of Catalyst on Pyrolysis Characteristics

**Figure 5:**
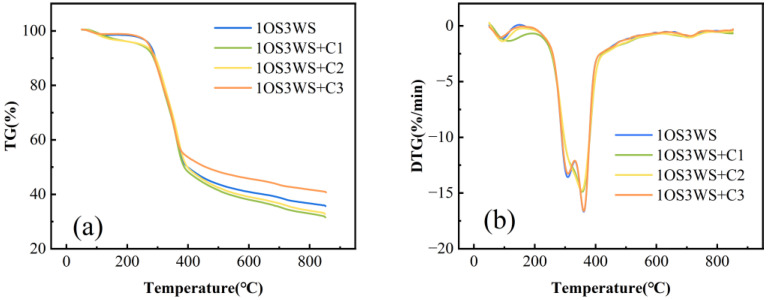
TG (**a**) and DTG (**b**) curves of the mixture of oily sludge and walnut shell under different catalyst conditions.

The pyrolysis curves of mixtures under different catalyst conditions are shown in Figure 5. As can be seen from the figure, the presence of ZSM-5 catalyst makes a big difference in the DTG_max_ of the mixture in the second stage. Compared with the sample without catalyst, the addition of a catalyst makes the initial decomposition temperature of the sample move in a lower direction, which is about 20 °C lower than that of the sample without catalyst, and the pyrolysis termination temperature also decreases. This may be attributed to the addition of the catalyst improving the pore structure of the mixture, making it easier for the volatile material to escape. In addition, with the increase of Zn content in the modified catalyst, the mass residual rate of the sample also decreases, which indicates that the modification of transition metal Zn ion on the molecular sieve effectively improves the catalytic activity of the catalyst.

### 3.3. Synergistic Effect Analysis of OS and WS Co-Pyrolysis

In order to illustrate the synergistic effect between the OS and WS samples, Δ*W* method was used for analysis. The equation is as follows:

(5) $$\Delta W=W_{exp}-x_{i}W_{i}$$

where *W_exp_* is the weight loss obtained from TG curve, *x_i_* is the mass percentage of each material in the mixture, and *W_i_* is the mass loss of each material under the same condition.

**Figure 6:**
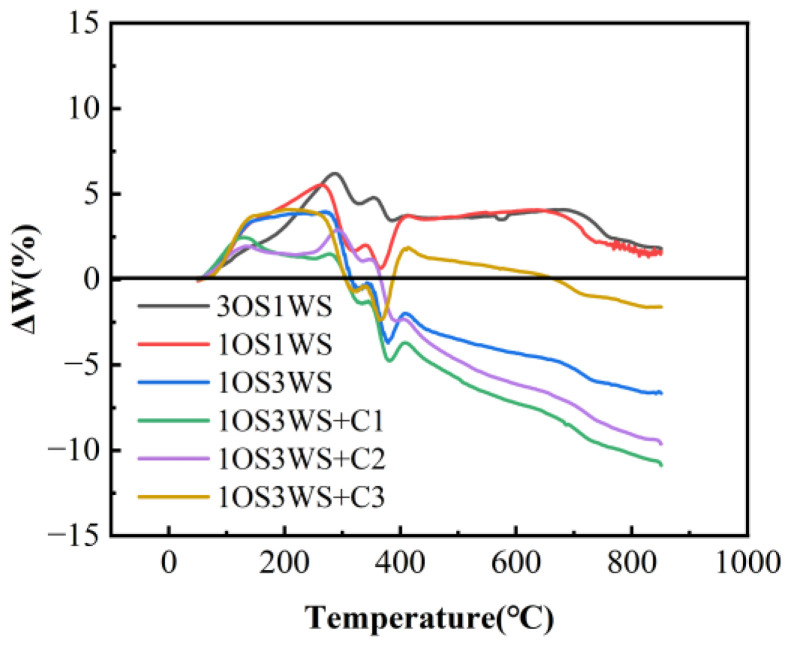
Variation of Δ*W* for the mixtures.

The variation of Δ*W* for the mixtures at 30 °C/min was shown in Figure 6. The negative and positive values of Δ*W* reflected the synergistic and inhibitory effects between the two raw materials, respectively. It can be observed that the value of Δ*W* is basically greater than zero when the temperature was less than 310 °C, which indicated that the presence of walnut shell inhibited the pyrolysis of oily sludge in the first stage of the co-pyrolysis of sludge and walnut shell. This was attributed to the fact that walnut shell was covered on the surface of oily sludge after decomposition and melting at low temperature, which inhibited the volatilization of light components in oily sludge. In the mixture of oily sludge and walnut shell, only the 1OS3WS group showed a synergistic effect in the second stage while the other two groups showed an inhibitory effect, which may be due to low biomass content, and could not promote the reaction between oily sludge and walnut shell. In the group 1OS3WS, Δ*W* reaches its minimum value around 376 °C and then becomes positive. In the samples with the catalyst added, the Δ*W* of mixture decreased significantly, showing a strong synergistic effect, which indicates that Zn-ZAM series catalysts have a better promotion effect on the pyrolysis of oily sludge and walnut shell.

### 3.4. Kinetic Analysis

Two typical model-free methods, the FWO and KAS methods, were used to estimate the activation energy of samples by using TG results at four different heating rates (10 °C/min, 20 °C/min, 30 °C/min, and 40 °C/min) [27]. The calculated activation energy and correlation coefficient (R2) are shown in Supplementary Table S1. When the conversion rate was between 0.1 and 0.9, the correlation coefficients were between 0.95 and 1.00, which indicated that the kinetic model was reliable. It was also found that there was no significant difference between the FWO and KAS models, ensuring the accuracy of the results.

**Figure 7:**
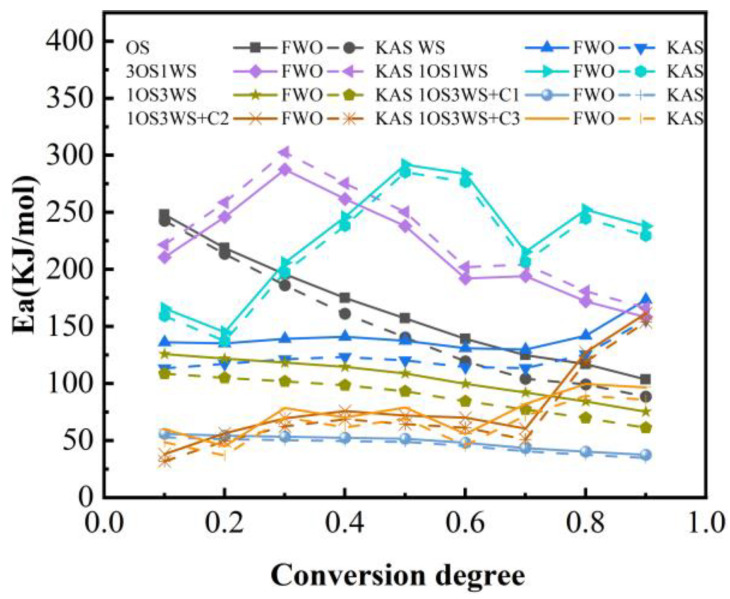
Variation of activation energy distribution of mixtures with degree of conversion.

The variation of activation energy distribution of mixtures with degree of conversion are shown in Figure 7. The activation energy E_a_ of oily sludge is from 164–151 kJ/mol. In the case of walnut shell pyrolysis alone, the mean values of FWO and KAS were 132.76 kJ/mol and 122.73 kJ/mol, respectively. The activation energy of oily sludge is higher than that of walnut shell, which means that oily sludge requires more energy than walnut shell to activate the pyrolysis process. The activation energies of OS, 3OS1WS, and 1OS1WS all fluctuate significantly with the conversion rate. This shows that the pyrolysis reaction of sludge is different from that of biomass, which is a multi-step reaction process. With the decrease of sludge ratio, the maximum activation energy changes to a larger conversion rate. This may be related to the degradation of the sample. The biomass is easy to decompose at low temperature and has low activation energy, while the sludge needs more energy to decompose at high temperature. The activation energy of 1OS3WS was the lowest among the three mixtures with an average value of 104.52 kJ/mol by FWO method and an average value of 88.89 kJ/mol by KAS method. In the process of catalytic co-pyrolysis, the apparent activation energies were lower than those of the co-pyrolysis samples without catalyst, indicating that the addition of catalyst promoted the pyrolysis reaction by reducing the E_a_ value.

### 3.5. Distribution of Pyrolysis Products

**Figure 8:**
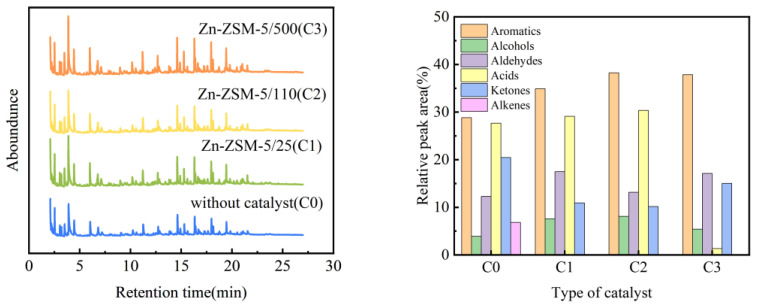
Total ion Chromatography (TIC) of products under different catalyst conditions.

The products containing a mixture of different catalysts in the pyrolysis plant was detected by GC/MS, and the total ion chromatogram is shown in Figure 8. As shown in the figure, the co-pyrolysis of oily sludge and walnut shell can produce a large number of aromatic hydrocarbons. After adding a Zn-modified ZSM-5 catalyst, the aromatic hydrocarbon content in the co-pyrolysis product was further increased. The specificity of ZSM-5 for aromatic hydrocarbons can be explained by its unique topology structure. ZSM-5 zeolite is rich in micropores, and its pore size is similar to the kinetic diameter of benzene. Therefore, aromatic hydrocarbons are more easily produced in ZSM-5 micropores. In the catalyst modified by the ZSM-5/25 molecular sieve, the content of Zn ions is more, and some olefin compounds will be produced. While when the catalyst modified by ZSM-5/500 molecular sieve has less Zn ion content, only a small amount of acid compounds will exist, thereby improving the quality of pyrolysis products. This is attributed to the increased density of acid sites with the increase of Zn content, which is conducive to the occurrence of hydrogen transfer reaction, resulting in higher aromatics yield [17,33]. The results show that ZSM-5 has special selectivity for aromatic hydrocarbons, and ZSM-5 molecular sieve with different silicon-aluminum ratios can have different effects on the co-pyrolysis products of oily sludge and biomass.

## 4. Conclusions

Thermogravimetric analysis was used to study the pyrolysis process and pyrolysis kinetics of the co-pyrolysis of oily sludge and walnut shell under different mixing ratios and catalysts with different Zn content. The distribution of catalytic cracking products was studied by PY-GC/MS. The results of the thermogravimetric analysis showed that the heating rate had little effect on the pyrolysis process. The addition of walnut shell can cover the oily sludge, which is beneficial to the completeness of the pyrolysis process. 1OS3WS showed the strongest synergy effect in co-pyrolysis, and the activation energy was lower than that of other mixtures. The presence of ZSM-5 zeolites contributes to this synergy effect by making the pyrolysis process complete faster. The activation energy after adding the catalyst is significantly lower than that of the samples without the catalyst, indicating that Zn-ZSM-5 promotes the co-pyrolysis process. The results of PY-GC/MS analysis showed that aromatics were mainly produced during the catalytic co-pyrolysis process, which may be caused by the unique pore structure of ZSM-5 zeolites.

## Figures and Tables

**Table 1:** Proximate analysis and element analysis of oily sludge and walnut shell.

Name	Proximate Analysis (wt%)				Ultimate Analysis (wt%)			
	Moisture	Ash	Volatile	Fixed Carbon	C	H	N	O
OS	5.94	77.32	15.84	6.84	52.72	11.35	1.66	34.27
WS	5.32	4.28	75	15.4	58.36	12.56	1.83	37.93

**Table 2:** Elemental composition of samples measured by ICP.

Sample	Elemental Composition (mmol/g)		
	Zn	Al	Si
ZSM-5/25	0	1.1	28.82
Zn-ZSM-5/25	0.22	1.22	32.17
ZSM-5/110	0	0.23	28.58
Zn-ZSM-5/110	0.08	0.25	31.43
ZSM-5/500	0	0.06	28.43
Zn-ZSM-5/500	0.03	0.06	31.44

**Table 3:** The main thermogravimetric parameters of different mixing ratios of oil sludge and walnut shell.

Samples	Stage 1				Stage 2				Stage 3				Final Mass Loss
	T_s_	T_e_	DTG_max_	T_max_	T_s_	T_e_	DTG_max_	T_max_	T_s_	T_e_	DTG_max_	T_max_	(%)
OS	101.4	292.8	3.7	191	316.2	537	0.5	442	617	775	1.5	687	15.2
OS:WS = 3:1	133	328	5.2	303	330	419	5.3	357	649	784	2.1	730	22
OS:WS = 1:1	151	330	9.6	307	332	422	10.2	360	648	793	2.1	732	31.6
OS:WS = 1:3	185	329	13.5	308	331	420	16.6	360	667	742	1.1	714	64.3
WS	175	386	18.6	352	--	--	--	--	--	--	--	--	72

T_s_: initial temperature; T_e_: end temperature; DTG_max_: maximum mass loss rate; T_max_: temperature corresponding to the maximum mass loss rate. Final mass loss: 100 minus the final residual rate of the TG curve.

## Data Availability

Not applicable.

## Supplementary Materials

The following supporting information can be downloaded at: https://www.mdpi.com/article/10.3390/ijerph20042841/s1, Figure S1. DTG curves of (a) OS, (b) WS, (c–e) their mixtures and (f–h) mixture with catalysts at different heating rates; Table S1. Pyrolysis kinetic parameters determined by FWO and KAS method.

## References

[1] Quan C., Zhang G., Xu L., Wang J., Gao N. (2022). Improvement of the pyrolysis products of oily sludge: Catalysts and catalytic process. J. Energy Inst..

[2] Mas F., Zhang G., Huang Q., Li J. (2021). Quantifying material flow of oily sludge in China and its implications. J. Environ. Manag..

[3] Wen Y., Xie Y., Jiang C., Li W., Hou Y. (2021). Products distribution and interaction mechanism during co-pyrolysis of rice husk and oily sludge by experiments and reaction force field simulation. Bioresour. Technol..

[4] Ma M., Xu D., Zhi Y., Yang W., Duan P., Wu Z. (2022). Co-pyrolysis re-use of sludge and biomass waste: Development, kinetics, synergistic mechanism and industrialization. J. Anal. Appl. Pyrolysis.

[5] Lin B., Huang Q., Chi Y. (2018). Co-pyrolysis of oily sludge and rice husk for improving pyrolysis oil quality. Fuel Process. Technol..

[6] Sun B., Huo J., Liu H., Che D., Guo S. (2023). Elucidation of synergistic effects in straw/sludge co-pyrolysis through gaseous product monitoring and biochar analysis. J. Energy Inst..

[7] Xu H., Cheng S., Hungwe D., Yoshikawa K., Takahashi F. (2022). Co-pyrolysis coupled with torrefaction enhances hydrocarbons production from rice straw and oil sludge: The effect of torrefaction on co-pyrolysis synergistic behaviors. Appl. Energy.

[8] Li Q., Gao Y., Ji G., Chen C., Li A. (2019). Evaluation of pyrolysis residue of oil sludge for recycling as bed material. Can. J. Chem. Eng..

[9] Song Q., Zhao H., Jia J., Zhang F., Wang Z., Lv W., Yang L., Zhang W., Zhang Y., Shu X. (2019). Characterization of the products obtained by pyrolysis of oil sludge with steel slag in a continuous pyrolysis-magnetic separation reactor. Fuel.

[10] Hakimian H., Pyo S., Kim Y.-M., Jae J., Show P.L., Rhee G.H., Chen W.-H., Park Y.-K. (2022). Increased aromatics production by co-feeding waste oil sludge to the catalytic pyrolysis of cellulose. Energy.

[11] Yang H., Yan R., Chen H., Lee D.H., Zheng C. (2007). Characteristics of hemicellulose, cellulose and lignin pyrolysis. Fuel.

[12] Chen C., Ling H., Qiu S., Huang X., Fan D., Zhao J. (2022). Microwave catalytic co-pyrolysis of chlorella vulgaris and oily sludge: Characteristics and bio-oil analysis. Bioresour. Technol..

[13] Hu G., Li J., Zhang X., Li Y. (2017). Investigation of waste biomass co-pyrolysis with petroleum sludge using a response surface methodology. J. Environ. Manag..

[14] Mortensen P.M., Grunwaldt J.D., Jensen P.A., Knudsen K.G., Jensen A.D. (2011). A review of catalytic upgrading of bio-oil to engine fuels. Appl. Catal. A Gen..

[15] Lai P.-C., Chen C.-H., Hsu H.-Y., Lee C.-H., Lin Y.-C. (2016). Methanol aromatization over Ga-doped desilicated HZSM-5. RSC Adv..

[16] Hou J., Zhong D., Liu W. (2022). Catalytic co-pyrolysis of oil sludge and biomass over ZSM-5 for production of aromatic platform chemicals. Chemosphere.

[17] Pinilla-Herrero I., Borfecchia E., Holzinger J., Mentzel U.V., Joensen F., Lomachenko K.A., Bordiga S., Lamberti C., Berlier G., Olsbye U. (2018). High Zn/Al ratios enhance dehydrogenation vs hydrogen transfer reactions of Zn-ZSM-5 catalytic systems in methanol conversion to aromatics. J. Catal..

[18] Müller S., Liu Y., Vishnuvarthan M., Sun X., van Veen A.C., Haller G.L., Sanchez-Sanchez M., Lercher J.A. (2015). Coke formation and deactivation pathways on H-ZSM-5 in the conversion of methanol to olefins. J. Catal..

[19] Pinilla-Herrero I., Olsbye U., Márquez-Álvarez C., Sastre E. (2017). Effect of framework topology of SAPO catalysts on selectivity and deactivation profile in the methanol-to-olefins reaction. J. Catal..

[20] Rojo-Gama D., Signorile M., Bonino F., Bordiga S., Olsbye U., Lillerud K.P., Beato P., Svelle S. (2017). Structure–deactivation relationships in zeolites during the methanol–to-hydrocarbons reaction: Complementary assessments of the coke content. J. Catal..

[21] Muller S., Liu Y., Kirchberger F.M., Tonigold M., Sanchez-Sanchez M., Lercher J.A. (2016). Hydrogen Transfer Pathways during Zeolite Catalyzed Methanol Conversion to Hydrocarbons. J. Am. Chem. Soc..

[22] Bjorgen M., Svelle S., Joensen F., Nerlov J., Kolboe S., Bonino F., Palumbo L., Bordiga S., Olsbye U. (2007). Conversion of methanol to hydrocarbons over zeolite H-ZSM-5: On the origin of the olefinic species. J. Catal..

[23] Zhou R., Huang B., Ding Y., Li W., Mu J. (2019). Thermal Decomposition Mechanism and Kinetics Study of Plastic Waste Chlorinated Polyvinyl Chloride. Polymers.

[24] Flynn J., Wall L.A. (1966). General Treatment of the Thermogravimetry of Polymers. J. Res. Natl. Bur. Stand. A Phys. Chem..

[25] Kissinger H. (1957). Reaction kinetics in differential thermal analysis. Anal. Chem..

[26] Flynn J.H. (1983). The isoconversional method for determination of energy of activation at constant heating rates. J. Therm. Anal..

[27] Muravyev N.V., Vyazovkin S. (2022). The Status of Pyrolysis Kinetics Studies by Thermal Analysis: Quality Is not as Good as It Should and Can Readily Be. Thermo.

[28] Vyazovkin S., Burnham A.K., Criado J.M., Pérez-Maqueda L.A., Popescu C., Sbirrazzuoli N. (2011). ICTAC Kinetics Committee recommendations for performing kinetic computations on thermal analysis data. Thermochim. Acta.

[29] Maziarka P., Anca-Couce A., Prins W., Ronsse F. (2022). A meta-analysis of thermo-physical and chemical aspects in CFD modelling of pyrolysis of a single wood particle in the thermally thick regime. Chem. Eng. J..

[30] Jin X., Teng D., Fang J., Liu Y., Jiang Z., Song Y., Zhang T., Siyal A.A., Dai J., Fu J. (2021). Petroleum oil and products recovery from oily sludge: Characterization and analysis of pyrolysis products. Environ. Res..

[31] Hochberg S.Y., Tansel B., Laha S. (2022). Materials and energy recovery from oily sludges removed from crude oil storage tanks (tank bottoms): A review of technologies. J. Environ. Manag..

[32] El Hamdouni Y., El Hajjaji S., Szabó T., Trif L., Felhősi I., Abbi K., Labjar N., Harmouche L., Shaban A. (2022). Biomass valorization of walnut shell into biochar as a resource for electrochemical simultaneous detection of heavy metal ions in water and soil samples: Preparation, characterization, and applications. Arab. J. Chem..

[33] Wu J.F., Yu S.M., Wang W.D., Fan Y.X., Bai S., Zhang C.W., Gao Q., Huang J., Wang W. (2013). Mechanistic insight into the formation of acetic acid from the direct conversion of methane and carbon dioxide on zinc-modified H-ZSM-5 zeolite. J. Am. Chem. Soc..

