# Supplementary material for: Investigation of Catalytic Co-Pyrolysis Characteristics and Synergistic Effect of Oily Sludge and Walnut Shell
Source: Int J Environ Res Public Health. 2023 Feb 6;20(4):2841. doi: 10.3390/ijerph20042841 (PMC9956203; doi:10.3390/ijerph20042841)
Supplement: Supplementary file 1 [file ijerph-20-02841-s001.zip › ijerph-2167797-SI.pdf]

Supplementary Information for

Investigation of catalytic co-pyrolysis characteristics and synergistic effect of oily sludge and walnut shell

Qinghong Li 1, Huan Yang 1,*, Ping Chen 2,3, Wenxue Jiang 2,3, Fei Chen 4, Xiaorong Yu 4,* and Gaoshen Su 1

1 School of Chemical and Environmental Engineering, Yangtze University, Jingzhou 434023, China; 202071245@yangtzeu.edu.cn (Q.L.); sugaoshen@163.com (G.S.)

2 Drilling and Production Engineering Technology Research Institute of CNPC Chuanqing Drilling

Engineering Co., Ltd., Chengdu 710018, China; chen_ping@cnpc.com.cn (P.C.); wenxue_j@cnpc.com.cn (W.J.)

3 National Engineering Laboratory for Exploration and Development of Low Permeability Oil and Gas Fields, Xi'an 710018, China

4 CCDC Chuangqing Downhole Technology Company, Xi'an 710018, China; cqjx_chenf@cnpc.com.cn (F.C.)

* Correspondence: yanghuan@yangtzeu.edu.cn (H.Y.); yxr_cjdx@163.com (X.Y.)

Figure

Figure S1 DTG curves of (a) OS, (b) WS, (c–e) their mixtures and (f–h) mixture with catalysts at different heating rates.

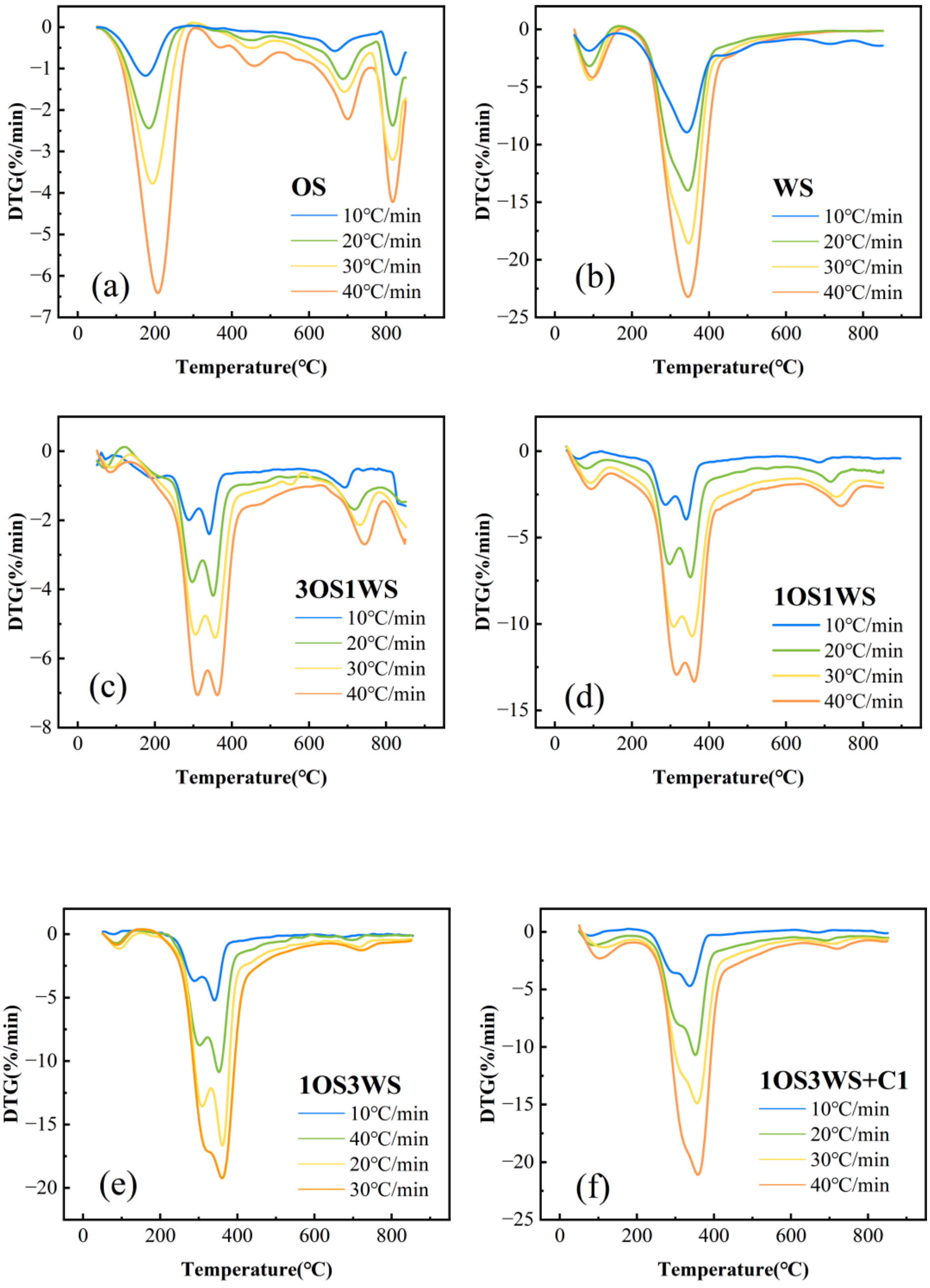

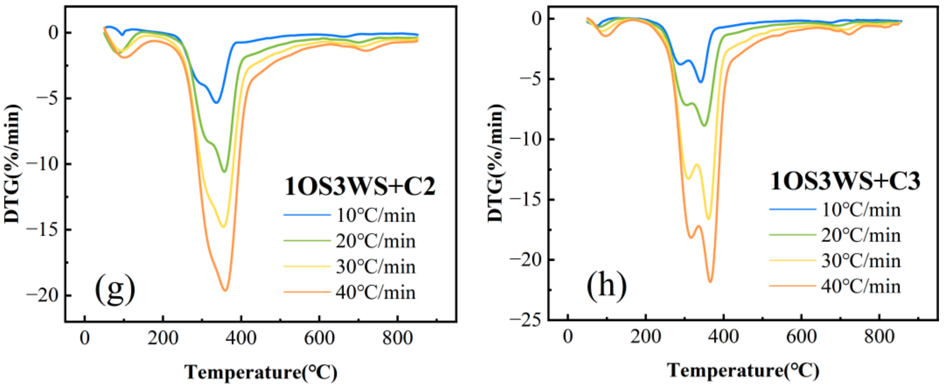

Table

Table S1. Pyrolysis kinetic parameters determined by FWO and KAS method.

Sample		FWO		KAS		Sample		FWO		KAS	
	α	E_a(KJ/mol)	R^2	E_a(KJ/mol)	R^2		α	E_a(KJ/mol)	R^2	Ea(KJ/mol)	R^2
OS	0.1	247.92	0.99	248.52	0.98	WS	0.1	66.18	0.99	113.25	0.98
	0.2	218.75	0.95	213.57	0.98		0.2	135.19	0.98	117.23	0.98
	0.3	195.74	0.99	186.03	0.98		0.3	139.10	0.95	121.25	0.98
	0.4	174.92	0.96	161.14	0.98		0.4	140.95	0.98	123.29	0.98
	0.5	157.12	0.98	140.33	0.98		0.5	137.40	0.98	120.21	0.98
	0.6	139.12	0.98	119.43	0.98		0.6	130.91	0.97	114.36	0.96
	0.7	124.85	0.98	104.18	0.99		0.7	129.74	0.95	113.58	0.95
	0.8	117.06	0.99	99.18	0.99		0.8	141.94	0.95	125.54	0.98
	0.9	103.51	0.99	88.50	0.99		0.9	173.43	0.96	155.89	0.98
Average		164.33		151.21				132.76		122.73	
3OS1WS	0.1	210.65	0.96	221.61	0.99	1OS1WS	0.1	165.84	0.98	159.28	0.95
	0.2	245.88	0.98	258.66	0.98		0.2	145.00	0.99	137.16	0.99
	0.3	287.60	0.98	302.55	0.98		0.3	205.84	0.98	197.43	0.98
	0.4	261.74	0.97	275.35	0.97		0.4	245.91	0.96	238.26	0.97
	0.5	237.94	0.99	250.31	0.99		0.5	291.79	0.99	285.19	0.99
	0.6	192.01	0.98	202.00	0.98		0.6	283.65	0.99	276.74	0.99
	0.7	194.07	0.98	204.16	0.99		0.7	215.34	0.98	206.60	0.99
	0.8	171.92	0.98	180.87	0.98		0.8	252.35	0.98	244.46	0.99
	0.9	158.11	0.98	166.33	0.98		0.9	237.91	0.99	229.57	0.99
Average		217.77		229.09		Average		227.07	0.99	219.41	
1OS3WS	0.1	125.81	0.99	108.57	0.98	1OS3WS+C1	0.1	55.91	1.00	52.76	1.00
	0.2	121.88	0.99	105.04	0.98		0.2	54.32	0.99	51.23	1.00
	0.3	118.30	0.99	101.83	0.98		0.3	53.36	1.00	50.36	1.00
	0.4	114.56	0.99	98.52	0.99		0.4	52.47	1.00	49.57	0.98
	0.5	108.78	0.99	93.23	0.99		0.5	51.57	1.00	48.82	0.99

	0.6	99.67	1.00	84.46	0.99		0.6	48.08	0.98	45.35	1.00
	0.7	92.12	0.99	77.30	0.99		0.7	43.47	1.00	40.69	1.00
	0.8	84.34	1.00	69.84	0.99		0.8	40.28	1.00	37.52	0.97
	0.9	75.26	0.99	61.18	0.99		0.9	37.50	1.00	34.81	1.00
Average		104.52		88.89		Average		48.55		45.68	
1OS3WS+ C2	0.1	38.13		31.91		1OS3WS+ C3	0.1	59.92	0.98	48.53	0.99
	0.2	56.42		49.39			0.2	45.13	0.99	36.93	0.98
	0.3	69.53		62.45			0.3	78.48	0.98	70.32	0.95
	0.4	75.83		68.64			0.4	70.22	0.98	61.53	0.98
	0.5	71.94		64.44			0.5	78.96	0.98	69.09	0.98
	0.6	70.17		61.24			0.6	55.82	0.98	45.64	0.99
	0.7	60.48		50.95			0.7	82.17	0.99	71.89	0.98
	0.8	127.34		119.12			0.8	99.60	0.99	89.27	0.99
	0.9	161.39		153.82			0.9	96.57	0.99	85.75	0.99
Average		81.25		73.55		Average		74.10		64.33	
